# Interpreting social determinants: Emergent properties and adolescent risk behaviour

**DOI:** 10.1371/journal.pone.0226241

**Published:** 2019-12-26

**Authors:** Chris Desmond, Janet Seeley, Candice Groenewald, Nothando Ngwenya, Kate Rich, Tony Barnett

**Affiliations:** 1 Centre for Rural Health, University of KwaZulu-Natal, Durban, South Africa; 2 Global Health and Development Department, London School of Hygiene and Tropical Medicine, London, United Kingdom; 3 Africa Health Research Institute, KwaZulu-Natal, Durban, South Africa; 4 Human and Social Development, Human Sciences Research Council, Durban, South Africa; 5 Department of Economics, University of Stellenbosch, Stellenbosch, South Africa; 6 Humanitarian and Conflict Response Institute, University of Manchester, Manchester, United Kingdom; University of Exeter, UNITED KINGDOM

## Abstract

A link between adversity, including low socio-economic status, and behaviours which carry health risks, such as alcohol consumption, has often been observed. The causes of this link are, however, poorly understood, making it difficult to explain why the association is often not linear and why there is so much variability between groups and individuals facing similar adversity. We investigate the use of the concept of emergent properties in explaining the link and its non-linear nature. `Emergent properties’ arise from the interaction of factors or items in a high-level system which, as a result, has qualities possessed by none of the individual factors. We apply a mixed methods approach to examine the association of an example emergent property, hope, and alcohol consumption among adolescents in a rural South African site. We found that among adolescents living in similar contexts, there was enough variance in reported levels of hope, that an association with alcohol use could be identified. This result is cause for optimism regarding the potential use of emergent properties in explaining variations in risk behaviour. Improving our measurement of emergent properties is perhaps the biggest challenge facing this approach. More work is needed to take further the task of identifying emergent properties capable of distilling the influence of lower level variables into single measures useful for analysis and policy purposes.

## Introduction: Pathways, interventions and emergent properties

Relationships between poor socio-economic conditions and a wide range of adverse health outcomes have been observed in numerous contexts. These relationships arise from direct health risks, stress from being at the lower end of the income/wealth distribution [1], and higher levels of risk behaviour [1, 2]. Here we explore: (a) aspects of why prevalence of risk behaviours is higher among people living in adverse environments, and (b) why the relationship between socio-economic status and risk behaviour is not linear.

There is considerable evidence of associations between risky environments and increased risk behaviour [3–6]. Although these associations are well established, the evidence is chequered regarding the *pathways* through which contexts influence health. This is an important *lacuna* as a better understanding of those pathways could facilitate the design of better interventions. However, the notion of “pathway” is itself problematic, adding a layer of largely unresolved complexity to any discussion of contextual influences on health status and outcomes.

In this paper we discuss pathways to risk behaviours among adolescents in South Africa while noting theoretical difficulties implicit in using “pathway” as a framing concept. In the absence of critical appraisal, the concept is all too often deployed in a mechanistic and linear manner implying a mechanistic theory of social, cultural and economic influences on the health status and rates of disease acquisition by individuals [3–8]. Such linearity is a conceptual simplification which may contribute to the design of simplistic interventions [9]. This research suggests that the nature of the association glossed in the term “pathway” may be clarified by introducing the theoretical idea of *emergent properties* [10–13] and that this clarification could contribute to improved design of interventions.

Different kinds of evidence suggests that local history, politics and culture are important components to be considered in the design of interventions intended to reduce the frequency of risk behaviours [14–17]. Generalised interventions which do not take account of such factors are less likely to succeed. It is likely that while such contextually sensitive approaches may appear expensive, cheaper generic interventions may waste money because, even if effective, they are sustainable only if and while funding is available [18]. Interventions which take account of contextual factors as experienced by the target population may turn out to be more cost effective and efficacious in the medium to long term [9], both because they may be more effective at changing behaviour, and because such changes may not require as much ongoing reinforcement. Such interventions may be said to take three broad forms: (a) major socio-economic restructuring via social movements and political changes; (b) social and economic reform of health and welfare systems; and (c) context specific manipulation of individual choice as in campaigns to achieve behaviour change. The 1958 Cuban revolution and the so-called “Washington Consensus” of the 1990s are contrasting examples of the first strategy; the mid-twentieth century health and welfare reforms in the United Kingdom, known as the “Welfare State”, are examples of the second; and (c) limited contextual interventions which demonstrate an understanding of the *choice architecture* within which individuals are able to make health choices [19] are examples of the third.

In this paper, we discuss changes enabling people to hope as a health intervention strategy engaging all these approaches in different degrees. If young people are to embrace hope for their individual and collective futures it is likely that interventions along all these dimensions are required but that interventions designed with choice architecture in mind may prove to be a pragmatic and feasible first step which can contribute to reforms at the other two levels.

Two deep intellectual influences frame our analysis. First are ideas from the work of the nineteenth century sociologist Emile Durkheim [20, 21]. He theorised that *social currents* were important in relation to the prevalence and incidence of suicide, an apparently individual act which he attempted to demonstrate was in fact intensely social, the outcome of the influence of *social currents* on the lives and life chances of individuals. Second is the very long tradition of thought which recognises that, in simple terms, the whole is greater than its parts–thus a higher-level system has qualities which are possessed by none of its individual parts. While a common example of such emergence is the snowflake where the crystalline structure is quite different from the molecular structure of water, so social distributions of power, income, wealth, taste, fashion and hope reflect processes of emergence. The framing theoretical idea of this research is recognition that individual behaviour is simultaneously limited by social structure and constitutes that structure. Sennett and Cobb’s *The Hidden Injuries of Class* [22] is an example of the application of this idea to class structure in the United States. The idea appears in sources as ancient as Aristotle [23] and since then and contemporarily in most fields of study from evolutionary theory to physics, biology and ecology [24–27].

To investigate the usefulness of this approach we examined how well our exemplar emergent property, hope, explains variance in alcohol use among adolescents in rural KwaZulu-Natal. We conceptualise hope as a distinct and operationalizable construct providing a way of measuring, via individuals and groups of individuals, indications of where ecologies of risk may be mapped as they affect individuals. The concept of hope may lead us to a better understanding of the pathways between individuals’ perspectives on their life worlds, social and economic conditions, and risk-taking behaviours. This perspective is dynamic, concerned with socio-economic and socio-cultural processes and offers a generalised entry point into diverse risk environments without homogenising this diversity into simple “pathways”.

Our study includes a qualitative component investigating young people’s understanding of hope and how it relates to risk. This informed the development of a measure of hope, which was then administered and its association with self-reported alcohol consumption examined. Measures of happiness and life-satisfaction were included as comparators. This allowed us to investigate how measures which reflect aspects of how individuals experience and interpret their environments compare, as predictors of risk behaviour, to a measure of socio-economic status, which provides a description of their environment, with no room for individual interpretation.

It is the potential that the interpretation, which occurs because of interactions between individual, group and context, could help explain differences in risk behaviour, which underlies the emergent properties approach. Hope is of interest because it speaks to how the individual sees their future from within their social structural position [28], their “life world”, a possibly important determinant of their present-day behaviour. Hope, happiness and life-satisfaction are likely to be linked to both individual and group narratives. That is, to how the social conditions are seen and understood through an interaction of individual and group. In this study, we are interested in the extent to which these measures provide an indication of how social determinants of health link to risk behaviour via these individual and group interactions, and to differences in interpretation. To clarify this problem, we posed the theoretical question: do variables such as hope, happiness and life satisfaction which seek to capture elements of individuals’ perceptions and experience of their structural contexts explain behaviour better than variables that seek to describe that context, for example an objective measure of socio-economic status?

## Study design

We adopted a mixed methods approach: (a) a qualitative component investigating risk behaviours and risk environments in relation to one example of an emergent property, hope [28–31]; (b) a quantitative component, which investigated the relationship between a measure of hope, developed from the qualitative findings, and differences in self-reported alcohol consumption.

In the qualitative component, discussions with adolescents focused on the meaning of hope, as understood by the target group, and to what extent they thought it captured aspects of their life-worlds [32, 33] relevant to explaining risk behaviour. This component included focus group discussions and individual interviews, details of which are provide below.

In the quantitative component, we gathered data on 500 adolescents to investigate the possibility that hope, measured initially as an index, was associated with self-reported risk behaviour. The design of this measure was informed by the qualitative work. The qualitative results informing the design and the survey methods are outlined below.

To provide a comparison we measured two other variables which capture how individuals see themselves and how they feel: these are happiness and life satisfaction. Both were included in the qualitative and quantitative components because, like hope, they may be “summary” indicators of individuals’ experience at the intersection of individual agency and social structure Barnett, Fournié [28]. We also compared the explanatory power of these variables with a measure of socio-economic status, an asset index.

### Study site

The study was conducted in KwaZulu-Natal in the Republic of South Africa at a site within the Demographic Surveillance Area (DSA) of the Africa Health Research Institute. The DSA is located near the market town of Mtubatuba, 250 km north of Durban in uMkhanyakude, a sub-district of Hlabisa. The population is largely Zulu-speaking and the area is predominantly rural, with an urban township and informal peri-urban settlements. Data were collected in 2017. A total of 8 research assistants employed an active recruitment strategy which included identifying potential participants during road shows (presentation of study to the community), in places where young people congregate such as the playgrounds, outside the shops and water taps. Snowball technique was also employed as individuals referred their friends or told the research assistants of other young people meeting the inclusion criteria. Before the consent and assent process, potential participants where asked where they wanted to be interviewed. All participants preferred to be interviewed in the privacy of their home with only the researcher and participant present in the space during the interview. Participants in the quantitative component were identified from the DSA database and interviewed at home.

### Ethics approach

The study was approved by the Biomedical Research Ethics Committee (BREC) of the University of KwaZulu-Natal (BE549/16) and the Ethics Committee of the Human Sciences Research Council (4/17/02/16). Informed consent was obtained from parents/primary caregivers for the participation of children in their care. Children were then asked if they agreed to participate. Both consent and assent were in writing. Information sheets, in Zulu, were read to caregivers and children, discussed and questions taken. These sheets were left with participants. They also contained contact details of the ethics committee, study PI, local coordinator and sources of support for adolescents.

### Qualitative methods

We investigated young people’s understandings and experiences of hope in relation to their perceptions and enactment of risk behaviours. We began with the Snyder Hope Scale as an exploratory starting point. While the scale had been validated for use in South Africa [34], we did not want to use it in the quantitative work without first ensuring that it captured a measure of hope relevant for our purposes. Concern has been raised that this measure frames hope as an individual construct rather than as a collectively shared concept, and may therefore may not be appropriate for use in South Africa [34]. Abler, Hill [35] have similarly raised concerns regarding the use of existing measures of hope in the South African context and have even proposed an alternative. Their suggested measure appears to perform well, but seems to capture mood rather than a driver of behaviour.

A guide, framed by the constituent items of the Snyder scale, was used to introduce discussions of hope with key informants in 30 Key Informant Interviews (KIIs) and four Focus Group Discussions (FGDs). The total sample comprised 53 young people, aged 15–17 years of age. We initially conducted 22 KIIs as per our sampling criteria, however the data and interaction with participants indicated some confusion regarding what we meant by hope. There was a challenge in translating the word ‘hope’ into Zulu as there are a number of alternative words. A further 7 KIIs were conducted using a revised topic guide to probe these different understandings and establish which of them respondents linked to risk behaviours.

Participants for the KIIs were purposively recruited to include a minimum of four 15–17 year-olds in each of the following categories: i) out of school youth’ ii) school going, iii) with resources such as flushable toilets/ piped water/ electricity, iv) with limited or no resources, v) parents with income, and vi) with parents with no income or with unstable income. For the group discussions, we sought four groups (6–8 participants) of young people also aged 15–17 years, two groups for males and two for females.

The semi-structured discussion guide was prepared in English and then translated into Zulu (Foxcroft and Roodt [36]. A participatory approach was adopted, engaging with young people using a lifeline drawing to promote discussion during the group discussions and interviews. This was used to unpack group ideologies and the social construction of the key concepts as well as the more subjective experiences and narratives of these concepts.

Interviews and FGDs were conducted in Zulu, audio-recorded, transcribed verbatim and translated. The transcripts were analysed using thematic analysis [37], facilitated by Atlas ti software (version 5). This study component of qualitative interrogation of the constructs and the measures provided us with an opportunity to revise the constructs for use in our survey.

The results of the qualitative analysis informed development of the quantitative measure of hope.

### Quantitative methods

We conducted a survey to examine the association between hope, happiness, life satisfaction and risk behaviours in a sample of adolescents, resident in the study site. Eligible participants were drawn at random from an existing database. We limited eligibility to a sub-region to limit costs. In keeping with ethical protocols, the guardians of eligible participants were approached at home and asked if they were willing to consent to the research team’s request to interview the child (15–17 years) in their care. Children were then approached and asked if they would agree to be interviewed.

The survey included age, gender, and a range of questions related to hope, happiness and life satisfaction [38] all rated on a Likert scale. The questions on hope, happiness and life satisfaction were selected based on the results of the qualitative component. Details of the measures are, therefore, outlined below alongside the qualitative results. The survey also included questions on whether respondents had ever used alcohol, *dagga* (cannabis), *tik* (methamphetamine), *buttons* (Mandrax), *whoonga* (heroin), ecstasy, or other drugs, whether the respondent was still using these substances, age at first use, and how regularly they used these substances [39]. Self-reports of substance use were very rare and as a result we only used the data on alcohol consumption.

Data from this survey were linked to data from the DSA on household and individual socio-economic characteristics, including data on sexual risk behaviour, an index constructed from household asset ownership, and the highest school grade attained. The data on sexual risk behaviour was of poor quality, with very high levels of non-response, and not used in the final analysis. The asset index was constructed using the first component extracted from principal component analysis (PCA), based on household ownership of a list of durable assets and the type of water source, cooking fuel, access to electricity, and toilet facilities [40]. A variable indicating whether a respondent was two or more school grades behind where they should be for their age was derived. This was based on their highest grade attained and their year of birth: the variable was selected with a view to capturing peer influences.

Following data collection and cleaning, analysis was undertaken in three steps. The first step involved basic descriptive analysis of the survey responses. The measures of hope, life satisfaction and happiness were summarised by individual items, as it was not obvious prior to data collection how items should be combined into a scale.

The second step of the analysis addressed this question, examining the approaches to using the hope, happiness and life satisfaction data, before choosing the third (as we explain below). The first involved simply summing the items. This is appropriate if there is a relatively common pattern across responses, such as when the Cronbach’s Alpha was high (above 0.7). The second approach considered was to construct an index using polychoric PCA (Poly-PCA); essentially a PCA for non-binary ordinal variables. The third option was to use the single item which most directly referred to the construct of interest.

The final stage of the analysis was a regression analysis with alcohol consumption as the dependent variable. Hope, happiness and life satisfaction were each included in two regressions without the other two, one with the asset index included and one without. This allowed us to examine whether they are associated with risk behaviour, and whether that association is independent of socio-economic status. Finally, a regression was conducted including hope, happiness, life satisfaction and assets. All regressions controlled for age and gender and whether the child was more than a year behind peers in school (as a control for peer effects on behaviour). In each case risk behaviour was a binary variable, therefore a logit regression was used, and odds ratios reported. All regressions were conducted in Stata.

## Results

### Qualitative results and the selection of quantitative tools

Hope, happiness and life satisfaction were all discussed with respondents in the qualitative phase. Respondents’ understanding of happiness and life satisfaction linked closely with the items in the associated scales. However, when we reviewed our findings on hope, and compared them to the Snyder scale, we found notable divergences. Moreover, analysis of the qualitative data revealed the complexity of the concept of hope, indicating that measurement may be a challenge, and that identifying the correct language around hope would be key.

The focus of the Snyder scale is on hope as an *individual* characteristic (part of the tradition of “positive psychology” [41]), a belief in what you can do. It did not appear to capture the idea of hope respondents linked to risk behaviour, which was a belief that things will improve. In particular, the scale focuses on how past experiences prepares individuals for current challenges, whereas respondents often focused on the future, on how to make it better than the past, and on their ability to influence that future, as factors shaping risk related decisions.

The adolescent respondents reported they were generally introduced to the notion of hope by teachers and parents during early schooling years. Hope was often thought of in relation to an object, person or goal; having hope in someone or a hope for something (a wish or a dream). Hope was also considered to be a belief or mind-set that was associated with positive future changes often articulated as a source of encouragement in the face of adversity, or a coping mechanism. Some also considered hope to resemble a character trait associated with self-belief, agency, goal setting and, to some extent, decision-making. For example, hopefulness, and conversely hopelessness, was perceived to be embedded within young people’s behaviours. Those who engaged in risk behaviours such as substance misuse were considered to have “no hope” and lack future aspirations. The absence of hope was seen to be a hindrance to goal attainment and achieving future success.

The two conceptualisations of hope, Snyder’s emphasis on individual capacity and the respondents’ emphasis on probable futures are related but sufficiently distinct that we did not include the Snyder Scale in the survey instrument. Rather, we developed a new hope scale derived from the qualitative data. This included eight questions related to hope. Table 1 lists the items and indicates the construct they were intended to capture. The items aimed to address the issues raised in the qualitative work relating to hope and risk behaviour and included risk related to individuals not considering the future, i.e. living in the moment; hope providing a way to cope with current adversity, preventing risk behaviour; and hope related to a future orientation and an associated sense of control. These were scored in interviews using a Likert Scale. The qualitative work also provided us with the appropriate language, in Zulu, to discuss hope in relation to each of these constructs, something we had initially struggled with in the focus groups and interviews.

**Table 1:** Hope items.

Survey items	Construct description	Support from qualitative findings
The future will take care of itself.	Disconnect with the future	“I can say that I wish one day, although I don’t know that day, even if I am no longer living in this community, to have development. Having good roads, [and people] being take care of in the township … (IDI 3)
I generally feel hopeful about my future.	Positive view of the future	“I can say that people must not lose hope in the community about things that they have heard or that they believe that are happening. Because hope is very important to human being’s life […] You need to trust that whatever that you are hoping for will happen or even if you heard that there is something that will come you have to **stay hoping** that it shall happen no matter how long it will take to happen.” (IDI 1)
Having hope helps me cope with day-to-day challenges.	Believing things will improve helps to manage present difficulties	
I have set long-term goals for my life.	Planning for the future	“It’s important to have your decisions! To take decisions that you make and starting **now** before you even reach matric level, so that you will know your life in future” (FGD 1)
I believe that if I work hard today, I can achieve my long term goals.	Self-belief, control over the future	“My plan is to study hard, be committed at school and maybe to pass with flying colours in matric- or even if it’s not flying colours, but to get **good results** so that I will be able to apply [to study at university]. So that they will see that **this person,** who applied here, knows what he wants!” (FGD 2)
I am confident that I can get the things that I hope for.		“I can say that it [*hope*] grows with years. Because my mother told me that when I was still young, although I wasn’t able to speak, that a man needs to trust himself in **anything** he does and he must never lose hope! Yeah, I can say that it grows with me because if I notice it really happens and even now hope keeps getting stronger in me. […] I think I was 7 years when my mother told me = I was starting at school […] that you have to hope for things and you will succeed!” (IDI 1) “What I understand about hope is that, as I am still at school, if my friend asks me about what I am hoping for or do, I have hope that I will pass at school. Because it is about **me** and I know **myself** that I can say I have hope that I **will** get a job, things like that. One **has to** have confidence because you are the one who takes actions! You know the end results.” (IDI 2)
It is easy for me to stick to my aims and accomplish my goals.		
I am confident that I could deal efficiently with unexpected events.	Self-belief in the face of uncertainty.	“I can say that you mustn’t lose your hope because you see that you don’t have a father and mother anymore, but I still have my sister and brothers. Let me make an example; maybe I have one older sister and the rest are young. You don’t have to lose hope! You need to tell yourself that ‘okay because I’m the old one here and I don’t have a father then maybe we are at the same age with my sister we will have to sit down both of us and discuss’. And discuss [and say]: ‘you know my sister if it’s like that, as you can see the situation, if we can be united on what we want to be [*support each other*], we **don’t have to lose hope**!’” (IDI 1)

### Quantitative results

#### Descriptive statistics

Data on 503 respondents were collected between 11 May 2017 and 1 September 2017: 261 males (52%) and 242 females (48%). Respondents ranged in age from 15 to 18 years, with an average age of 16.48 (i.e. 16 years and 174 days). A total of 121 (24.40%) respondents were more than two grades behind the appropriate grade for their age in school. Table 2 provides a summary of the basic demographic characteristics.

**Table 2:** Sample characteristics.

	Number	Percentage
Average age	16.48	
Male	261	51.89
Female	242	48.11
More than one grade behind in school	245	49.40
More than two grades behind in school	121	24.40

Table 3 summarises the responses to the eight hope items. The results indicate a high level of agreement with the positively framed items and a high level of disagreement with the one negatively framed item.

**Table 3:** Hope responses, by item.

	Strongly Disagree	Disagree	Neutral	Agree	Strongly Agree
I generally feel hopeful about my future	1%	1%	3%	49%	48%
The future will take care of itself.	47%	3%	12%	24%	14%
Having hope helps me cope with day-to-day challenges.	1%	1%	2%	57%	39%
I have set long-term goals for my life.	7%	1%	4%	53%	35%
I believe that if I work hard today, I can achieve my long term goals	1%	0%	2%	52%	45%
I am confident that I can get the things that I hope for.	6%	0%	7%	50%	36%
It is easy for me to stick to my aims and accomplish my goals.	10%	0%	7%	49%	34%
I am confident that I could deal efficiently with unexpected events.	9%	0%	7%	52%	32%

The responses to the life satisfaction scale, by item, are reported in Table 4; the responses are positively skewed for the first three items. The final two items do not follow the same pattern. It is not immediately clear how to reconcile being satisfied with life with not having got what you want and indicating that if you had the chance you would change the past. The first of the negative responses could be interpreted as reflecting the age of the respondents–‘So far I have gotten the important things I want in life’ being disagreed with because they see these things in their future. However, the final item suggests that many respondents are not satisfied with how their life has gone. This is difficult to reconcile with their reported satisfaction overall.

**Table 4:** Life satisfaction responses, by item.

	Strongly Disagree	Disagree	Neither agree nor disagree	Agree	Strongly Agree
In most ways my life is close to my ideal.	9%	6%	11%	45%	28%
The conditions of my life are excellent.	6%	4%	6%	51%	32%
I am satisfied with my life.	7%	4%	5%	54%	30%
So far I have gotten the important things I want in life.	32%	6%	9%	37%	17%
If I could live my life over, I would change almost nothing.	30%	1%	3%	43%	23%

Table 5 summarises the item responses to the happiness questions. Most respondents reported being happy or extremely happy. When asked to compare themselves to their peers a larger majority reported the same,. However, less than half reported being happy or extremely happy in the last month.

**Table 5:** Happiness responses, by item.

	Extremely unhappy	Unhappy	Neutral	Happy	Extremely Happy
In general, I consider myself:	1%	2%	21%	46%	30%
Compared to most of my peers, I consider myself:	2%	4%	18%	40%	37%
Thinking about the last month, how would you describe your happiness?	11%	5%	42%	21%	22%

There was very little self-reported drug use, and low self-reported alcohol use, see Table 6. It is possible that respondents may not have been comfortable admitting to these behaviours.

**Table 6:** Substance use.

	No	Yes
Ever used:		
Alcohol	72%	28.2%
Dagga (Zol)	100%	0.4%
Tik (Meth)	100%	0.2%
Buttons (Mandrax)	100%	0.0%
Whoonga (Heroin)	100%	0.2%
E (Ecstasy	100%	0.0%
Other drugs	100%	0.0%

### Regression results: The association between hope and alcohol consumption

We conducted a regression analysis to examine the association between reporting alcohol use and the exemplar emergent property, hope. For comparison, the association with happiness and life satisfaction is also examined. In each case, the strength of that association is compared to the association between socio-economic status, as measured by the asset index, and alcohol use.

Before conducting the regression analyses, we had first to select the most appropriate of the three approaches to using the hope, happiness and life satisfaction data. The first approach, simply summing the responses, would have been appropriate had there been relatively common pattern across responses. The Cronbach Alphas suggest that this is not the case; none was above 0.7 (Hope 0.65; Life Satisfaction 0.64; Happiness 0.53). The second approach, constructing indices using Poly-PCA, was conducted. The eigen values (see S3 Table) suggested that it was appropriate to use only the first component as the index for each construct. However, given the patterns of responses, and the associated component loadings (see S4 Table) the indices are difficult to interpret. It appears that not all the items in each measure are measuring elements of a common underlying construct. The third option, i.e. to use the single item which most directly referred to the construct of interest, is more straightforward to interpret than the Poly-PCA. We do not have to make sense of component loadings in the indices. We therefore settled on the use of a single item. While we focus on the results using this third option, all analyses are repeated using the indices created with poly-PCA and these regressions are reported in the supporting information. The following single items in the regression analysis.

- Hope: I generally feel hopeful about my future, scored 1–5 by strongly disagree, disagree, neither agree nor disagree, agree, strongly agree.
- Life satisfaction: I am satisfied with my life, scored 1–5 by strongly disagree, disagree, neither agree nor disagree, agree, strongly agree.
- Happiness: In general, I consider myself (extremely unhappy, unhappy, neutral, happy, extremely happy), scored 1–5.

The use of a direct single item measure has positive and negative implications. It allows for variation in individual interpretations of hope, happiness and life satisfaction. Thus, it may better capture how individuals feel. However, we are no longer imposing an interpretation, as would be the case if we used a combination of multiple items, which makes interpreting the results more difficult.

The regression results using the single item measure of hope are reported in Table 7. Three regressions were conducted. For each test the dependent variable is whether the respondent reported any alcohol use. All regressions include age, gender and whether the adolescent is more than a year behind in school as controls. The difference between the regressions is that the first includes the asset index, the second includes the measure of hope, and the third assets and hope. The table reports odds ratios. A ratio of more than 1 indicates a positive correlation with the probability of reporting alcohol consumption, less than 1 a negative correlation and 1 indicates no association. In all three regressions, being older and male are found to be positively associated with alcohol use, as would be expected. Being two or more grades behind in school was negatively associated (odds ratio less than 1) with alcohol consumption, as expected, reflecting a younger peer group. However, the relationship was weak and only significant in models 1 and 3 and then only at the 10% level.

**Table 7:** Alcohol use: Logit regressions including hope, assets, and hope and assets, odds ratios reported.

VARIABLES	(1)	(2)	(3)
Age	1.407*** (1.147–1.726)	1.388*** (1.137–1.695)	1.433*** (1.165–1.763)
Male	1.908*** (1.244–2.925)	1.863*** (1.231–2.819)	1.868*** (1.215–2.873)
Two or more grades behind in school	0.656 (0.396–1.087)	0.685 (0.419–1.118)	0.621* (0.373–1.034)
Asset index	0.944 (0.848–1.052)		0.934 (0.838–1.041)
I generally feel hopeful about my future		0.734** (0.539–0.998)	0.694** (0.504–0.955)
Constant	0.001*** (0.000–0.032)	0.005*** (0.000–0.168)	0.004*** (0.000–0.149)
Observations	472	496	472

Confidence intervals in parentheses

*** p<0.01,

** p<0.05,

* p<0.1

The result of primary interest is the significant negative correlation between hope and alcohol use (an odds ratio of less than 1). By comparison, there was no significant association between the household asset index and alcohol consumption. Including both hope and assets in the regression does not change the result for either, suggesting that they are not measuring similar things, i.e. hope is not simply a correlate of socio-economic status.

Table 8 reports the results of five regressions. The first regression is the same as the first regression in the previous table, it is repeated to facilitate comparison. Regressions two and three include life satisfaction, and life satisfaction and assets respectively. Regressions four and five include happiness, and happiness and assets, respectively.

**Table 8:** Alcohol use: Logit regression, including happiness and life satisfaction, with or without assets, odds ratios reported.

VARIABLES	(1)	(2)	(3)	(4)	(5)
Age	1.407*** (1.147–1.726)	1.357*** (1.112–1.655)	1.398*** (1.139–1.716)	1.338*** (1.095–1.635)	1.380*** (1.122–1.698)
Male	1.908*** (1.244–2.925)	1.938*** (1.278–2.940)	1.951*** (1.267–3.002)	1.909*** (1.259–2.895)	1.905*** (1.239–2.930)
Two or more grades behind in school	0.656 (0.396–1.087)	0.713 (0.436–1.166)	0.654 (0.393–1.090)	0.684 (0.417–1.122)	0.631* (0.378–1.054)
Asset index	0.944 (0.848–1.052)		0.949 (0.851–1.058)		0.942 (0.845–1.050)
I am satisfied with my life		0.787*** (0.657–0.943)	0.806** (0.668–0.972)		
In general, I consider myself (how happy)				0.704*** (0.556–0.891)	0.712*** (0.560–0.906)
Constant	0.001*** (0.000–0.032)	0.005*** (0.000–0.142)	0.003*** (0.000–0.090)	0.010*** (0.000–0.323)	0.006*** (0.000–0.214)
Observations	472	496	472	496	472

Confidence intervals in parentheses

*** p<0.01,

** p<0.05,

* p<0.1

Age and gender were again significant in all regressions with the expected relationship: older males more likely to report alcohol consumption. The asset index was not significant in any of the regressions. The single item measures of happiness and life satisfaction both led to significant odds ratios below one, implying a negative association between them and the probability of alcohol consumption.

The final regression conducted is reported in Table 9. It includes the standard controls plus assets, hope, life satisfaction and happiness. Again, the associations between the probability of reporting alcohol use and age and gender were as expected. The asset index again was not associated with alcohol use. With the measures of hope, happiness and life satisfaction all included in the same regression, only happiness showed a significant association with probability of alcohol use.

**Table 9:** Alcohol use regression, including assets, hope, happiness and life satisfaction.

VARIABLES	(1)
Age	1.400*** (1.135–1.727)
Male	1.903*** (1.232–2.942)
Two or more grades behind in school	0.614* (0.366–1.029)
Asset index	0.938 (0.841–1.047)
I generally feel hopeful about my future	0.746* (0.537–1.035)
I am satisfied with my life	0.850 (0.698–1.035)
In general, I consider myself (how happy)	0.771** (0.598–0.994)
Constant	0.023* (0.000–1.048)
Observations	472

Confidence intervals in parentheses

*** p<0.01,

** p<0.05,

* p<0.1

## Limitations

The study is hindered by the low rates of reporting of risk behaviours. In the case of sexual behaviour, there was too much missing data for the variable to be used. In addition, the response rates for drug use were too low for the variable to be used. It is possible that these rates are correct but given established rates of substance use among adolescents in KwaZulu-Natal, this is unlikely.

The low rates of reporting left us with usable data on only one risk behaviour, alcohol use. There is a concern that this too suffered from reporting bias. If there is reporting bias, and it is associated with any of the measures, then our results are biased. However, for the bias to have led us to erroneous conclusions, it would have to be because those who were more hopeful (happy and satisfied), were more likely to conceal alcohol consumption, which seems unlikely. Similarly, if those with lower SES systematically concealed alcohol consumption more often, the bias would have implications for our results. There is no reason to expect this to be the case. If the misreporting was not associated with our variables, the bias is not a concern for our conclusions.

This is a cross-sectional study and there is a possibility of reverse causation. It is possible that alcohol consumption makes people less hopeful or unhappy. Alternative longitudinal approaches to data collection will be required to address this concern in the future.

The study population was relatively homogeneous in terms of socio-economic status. The lack of variance in socio-economic status may explain the weakness of the association between the asset index and alcohol consumption. However, it is interesting that despite the population being homogeneous, there was sufficient variation in hope, happiness and life satisfaction measures to identify a relationship with alcohol consumption.

## Discussion

Studies of the social determinants of health have repeatedly shown that there are strong associations between adverse social conditions and certain risk behaviours [42]. Little attention, however, has been paid to the role of individual decision-making in linking context and behaviour. There are discussions of empowerment, and the extent to which adverse conditions reduce people’s sense that they can do something about their situation [42]. However, even these are based on a thin conceptualisation of the people involved. Little or no space is given to trying to understand how individuals and groups understand their environment, how they interact with and change their environment, and how these understandings and interactions shape behaviour.

In this analysis we recognise that “the social” and “social structure” consists of a complex set of fluid interactions between emergent properties of the socio-economic-cultural spaces within which we lead our lives [43–45]. It is this complexity which engages with the idea that: (a) a “pathway” is not a constant, mechanical relationship, but rather reflects the generation, alignment and interaction of emergent properties with particular places and times; and thus (b) any interventions should engage with the shifting nature of the terrains in which they are located.

A view of individuals which focuses on the importance of their (fluid) interpretation of the environment, and how this shapes their behaviour, requires a focus on what characterises that environment and what influences people’s understanding of those characteristics. This in turn highlights the importance of both group influences and individual variation. The environment and different understandings of that environment are shaped by groups. Yet not all members of groups share a common understanding. These are complex conceptual and logical issues. The approach through emergent properties is a step towards a fuller conceptualisation of risk behaviours and their link to the social individual. It does this by providing a way to investigate the role of interpretation in linking environment to behaviour. It allows us to ask if different interpretations help explain different responses, i.e. why the relationship between environment and risk behaviour is not linear or uniform. It can be used to examine group interpretations of the environment, and individual variation within groups.

In this study we focused on a relatively homogeneous group. Therefore the analysis, has focused on individual variation within the group. To facilitate this examination of individual variation, we first had to examine the ways in which the group understands the links between context and behaviour. Using hope as an exemplar of an emergent property, we worked with young people to develop a fuller understanding of what hope means to them, and whether they saw links between hope and risk behaviour.

The qualitative analysis suggested that young people in the study area believed that there was a link between hope and risky behaviours. On one hand, they emphasised a link between giving up hope and engaging in risky health-related behaviours; on the other hand, they emphasised the link between hope and future orientation and low-risk health behaviours.

Through our qualitative component we found that the concept of hope was difficult to translate into Zulu, as it could be understood in several different ways. As a result, we had to refine the initial scale significantly. The quantitative results suggest that within our study population the level of hope reported by an individual is more strongly associated with their risk behaviour than is their socio-economic status. Thus, regression analysis showed a statistically significant negative relationship between hope and risk behaviour. We did not find the same for the asset index. However, neither measure explained much of the variance in risk behaviour. This lack of explanatory power suggests that other factors which explain individual differences in alcohol consumption were not captured. This result is grounds for cautious optimism: optimism stems from the exemplar out-performing a well-established measure of socio-economic status, known to be associated with risk; caution, from how weakly both hope and the asset index were associated with alcohol consumption.

The regression results indicated a negative relationship between hope and the probability of alcohol consumption, i.e. higher levels of reported hope were associated with a lower probability of reporting alcohol consumption. Similarly, the direct questions on happiness and life satisfaction had significant negative associations with alcohol use. The asset index, which was used as a measure of socio-economic status was, however, not associated with alcohol use.

The lack of association between assets and alcohol consumption may reflect homogeneity of socio-economic status within the sample. It is noteworthy that even among adolescents living in similar contexts, there was enough variance in reported levels of hope, that an association with alcohol use could be identified. This result is cause for optimism regarding the potential use of emergent properties in explaining variations in risk behaviour.

Improving measurement of emergent properties is perhaps the biggest challenge facing this approach. The regression results suggest that there is significant overlap between hope, happiness and life satisfaction. When all are included in the same regression only happiness is significantly associated with alcohol consumption. More work is needed to take further the task of identifying emergent properties capable of distilling the influence of lower level variables into single measures useful for analysis and policy purposes. A feasible next step may be to examine the association between risky health behaviours and combinations of emergent properties. A single emergent property may not capture enough to adequately explain differences in risk. Combinations are likely to cluster, leading to emergent narratives, with these narrative framing and influencing risk behaviours. The possible importance of narratives was evident in the patterns of response to the various scales. It helps to explain how individuals may be happy and satisfied, yet not happy with the last month or satisfied with how things have gone. Firstly, people may need time to process recent experiences into their narratives. Secondly, once assimilated, people’s current narratives may differ from their past experiences [12]. Understanding the development of emergent narratives as accounts of how people experience the influence of factors constraining their agency which may be summarised through emergent properties like hope may be important for further understanding of how emergent properties can be used in developing policies relating to health risk behaviours.

## Supporting information

S1 Table Logit regression analysis including assets (1), hope index (2), hope index and assets (3), odds ratios reported. (DOCX)

S2 Table Logit regression analysis including assets (1), life satisfaction index (2), life satisfaction index and assets (3), happiness index (4), happiness index and assets (5), odds ratios reported. (DOCX)

S3 Table Eigen values of polychoric PCA for hope index. (DOCX)

S4 Table Loadings for hope index. (DOCX)

S5 Table Odds ratios from regression analysis including both the first and second component of hope index constructed using poly-PCA. (DOCX)
