# Supplementary material for: Interpreting social determinants: Emergent properties and adolescent risk behaviour
Source: PLoS One. 2019 Dec 26;14(12):e0226241. doi: 10.1371/journal.pone.0226241 (PMC6932798; doi:10.1371/journal.pone.0226241)
Supplement: S1 Table — (DOCX) [file pone.0226241.s001.docx]

**SUPPORTING INFORMATION**

**Table S1. Logit regression analysis including assets (1), hope index (2), hope index and assets (3), odds ratios reported**

| VARIABLES | (1) | (2) | (3) |
| --- | --- | --- | --- |
|  |  |  |  |
|  |  |  |  |
| Age | 1.407*** | 1.367*** | 1.405*** |
|  | (1.147 - 1.726) | (1.120 - 1.668) | (1.144 - 1.726) |
| Male | 1.908*** | 1.888*** | 1.910*** |
|  | (1.244 - 2.925) | (1.250 - 2.851) | (1.245 - 2.928) |
| Two or more grades behind in school | 0.656 | 0.721 | 0.658 |
|  | (0.396 - 1.087) | (0.443 - 1.175) | (0.396 - 1.093) |
| Asset index | 0.944 |  | 0.944 |
|  | (0.848 - 1.052) |  | (0.848 - 1.052) |
| Hope index (poly-PCA) |  | 1.000 | 1.007 |
|  |  | (0.889 - 1.125) | (0.892 - 1.137) |
| Constant | 0.001*** | 0.002*** | 0.001*** |
|  | (0.000 - 0.032) | (0.000 - 0.046) | (0.000 - 0.033) |
|  |  |  |  |
| Observations | 472 | 496 | 472 |

Confidence intervals in parentheses

*** p<0.01, ** p<0.05, * p<0.1

Tables S1 shows the results of the standard regressions. It includes indices of hope as continuous variable. Indices were constructed using polychoric TCA. For hope, the index was insignificant.
