# Supplementary material for: Interpreting social determinants: Emergent properties and adolescent risk behaviour
Source: PLoS One. 2019 Dec 26;14(12):e0226241. doi: 10.1371/journal.pone.0226241 (PMC6932798; doi:10.1371/journal.pone.0226241)
Supplement: S2 Table — (DOCX) [file pone.0226241.s002.docx]

**SUPPORTING INFORMATION**

**Table S2. Logit regression analysis including assets (1), life satisfaction index (2), life satisfaction index and assets (3), happiness index (4), happiness index and assets (5), odds ratios reported**

| VARIABLES | (1) | (2) | (3) | (4) | (5) |
| --- | --- | --- | --- | --- | --- |
|  |  |  |  |  |  |
|  |  |  |  |  |  |
| Age | 1.407*** | 1.358*** | 1.398*** | 1.333*** | 1.374*** |
|  | (1.147 - 1.726) | (1.114 - 1.655) | (1.139 - 1.716) | (1.092 - 1.629) | (1.118 - 1.690) |
| Male | 1.908*** | 1.917*** | 1.928*** | 1.912*** | 1.913*** |
|  | (1.244 - 2.925) | (1.266 - 2.901) | (1.255 - 2.962) | (1.264 - 2.892) | (1.247 - 2.937) |
| Two or more grades behind in school | 0.656 | 0.716 | 0.655 | 0.711 | 0.654 |
|  | (0.396 - 1.087) | (0.440 - 1.167) | (0.395 - 1.088) | (0.437 - 1.159) | (0.394 - 1.085) |
| Asset index | 0.944 |  | 0.949 |  | 0.946 |
|  | (0.848 - 1.052) |  | (0.852 - 1.058) |  | (0.849 - 1.054) |
| Life satisfaction index (poly-PCA) |  | 0.871* | 0.877* |  |  |
|  |  | (0.755 - 1.005) | (0.757 - 1.016) |  |  |
| Happiness index (poly-PCA) |  |  |  | 0.843* | 0.849* |
|  |  |  |  | (0.709 - 1.002) | (0.711 - 1.014) |
| Constant | 0.001*** | 0.002*** | 0.001*** | 0.003*** | 0.002*** |
|  | (0.000 - 0.032) | (0.000 - 0.050) | (0.000 - 0.035) | (0.000 - 0.070) | (0.000 - 0.049) |
|  |  |  |  |  |  |
| Observations | 472 | 496 | 472 | 496 | 472 |

Confidence intervals in parentheses

*** p<0.01, ** p<0.05, * p<0.1

Tables S2 shows the results of the standard regressions. It includes indices of life satisfaction and happiness as continuous variable. Indices were constructed using polychoric TCA. For life satisfaction and happiness the indices were only significant at the 10% level.
