# Supplementary material for: Interpreting social determinants: Emergent properties and adolescent risk behaviour
Source: PLoS One. 2019 Dec 26;14(12):e0226241. doi: 10.1371/journal.pone.0226241 (PMC6932798; doi:10.1371/journal.pone.0226241)
Supplement: S3 Table — (DOCX) [file pone.0226241.s003.docx]

**SUPPORTING INFORMATION**

**Table S3. Eigen values of polychoric PCA for hope index**

| k | Eigenvalues | Proportion explained | Cum. explained |
| --- | --- | --- | --- |
| 1 | 4.306788 | 0.538348 | 0.538348 |
| 2 | 0.985340 | 0.123167 | 0.661516 |
| 3 | 0.674164 | 0.084271 | 0.745786 |
| 4 | 0.544939 | 0.068117 | 0.813904 |
| 5 | 0.442581 | 0.055323 | 0.869226 |
| 6 | 0.417993 | 0.052249 | 0.921476 |
| 7 | 0.353561 | 0.044195 | 0.965671 |
| 8 | 0.274635 | 0.034329 | 1.000000 |

Only the first principal component has an eigenvalue above 1.
