# Supplementary material for: Interpreting social determinants: Emergent properties and adolescent risk behaviour
Source: PLoS One. 2019 Dec 26;14(12):e0226241. doi: 10.1371/journal.pone.0226241 (PMC6932798; doi:10.1371/journal.pone.0226241)
Supplement: S4 Table — (DOCX) [file pone.0226241.s004.docx]

**SUPPORTING INFORMATION**

**Table S4. Loadings for hope index**

|  |  | Coef. 1 | Coef. 2 | Coef. 2 |
| --- | --- | --- | --- | --- |
| I generally feel hopeful about my future | 1  2  3  4  5 | -1.060938  -0.919430  -0.786502  -0.396922  0.153144 | 0.436795 0.378535  0.323808 0.163415  -0.063050 | -1.453621  -1.259736  -1.077609  -0.543834  0.209826 |
| The future will take care of itself. | 1  2  3  4  5 | -0.182542  -0.097177  -0.081352  -0.046325  0.096790 | -1.193454  -0.635340  -0.531877  -0.302873  0.632813 | -0.424540  -0.226006  -0.189202  -0.107739  0.225107 |
| Having hope helps me cope with day-to-day challenges. | 1  2  3  4  5 | -1.102131  -0.944018  -0.864282  -0.420873  0.198610 | 0.401996 0.344325  0.315242 0.153511  -0.072442 | -0.014219  -0.012179  -0.011151  -0.005430  0.002562 |
| I have set long-term goals for my life. | 1  2  3  4  5 | -0.772348  -0.626080  -0.581717  -0.319448  0.185090 | 0.469417  0.380518  0.353556  0.194154  -0.112494 | -1.068806  -0.866394  -0.805003  -0.442065  0.256135 |
| I believe that if I work hard today, I can achieve my long term goals | 1  2  3  4  5 | -1.181393  -  -0.979450  -0.473381  0.192674 | 0.282085  -  0.233867  0.113031  -0.046005 | -0.178368  -  -0.147878  -0.071472  0.029090 |
| I am confident that I can get the things that I hope for. | 1  2  3  4  5 | -0.878085  -0.723639  -0.645142  -0.350576  0.204772 | -0.130376  -0.107444  -0.095789  -0.052053 0.030404 | 0.619990  0.510940  0.455515  0.247532  -0.144584 |
| It is easy for me to stick to my aims and accomplish my goals. | 1  2  3  4  5 | -0.723109  -0.575466  -0.524788  -0.294271  0.191840 | -0.503318  -0.400551  -0.365277  -0.204827  0.133530 | 1.016096  0.808632  0.737421  0.413503  -0.269569 |
| I am confident that I could deal efficiently with unexpected events. | 1  2  3  4  5 | -0.779018  -0.628083  -0.568267  -0.308419  0.204465 | 0.092331 0.074442  0.067352  0.036555  -0.024234 | 0.713168  0.574992  0.520232  0.282348  -0.187182 |
