# Supplementary material for: Interpreting social determinants: Emergent properties and adolescent risk behaviour
Source: PLoS One. 2019 Dec 26;14(12):e0226241. doi: 10.1371/journal.pone.0226241 (PMC6932798; doi:10.1371/journal.pone.0226241)
Supplement: S5 Table — (DOCX) [file pone.0226241.s005.docx]

**SUPPORTING INFORMATION**

**Table S5. Odds ratios from regression analysis including both the first and second component of hope index constructed using poly-PCA**

| VARIABLES | (1) | (2) | (3) |
| --- | --- | --- | --- |
|  |  |  |  |
|  |  |  |  |
| Age | 1.367*** | 1.366*** | 1.366*** |
|  | (1.120 - 1.668) | (1.120 - 1.665) | (1.119 - 1.668) |
| Male | 1.888*** | 1.842*** | 1.842*** |
|  | (1.250 - 2.851) | (1.217 - 2.789) | (1.216 - 2.789) |
| Two or more grades behind in school | 0.721 | 0.720 | 0.719 |
|  | (0.443 - 1.175) | (0.442 - 1.172) | (0.440 - 1.174) |
| Hope poly-PCA component 1 | 1.000 |  | 0.998 |
|  | (0.889 - 1.125) |  | (0.886 - 1.123) |
| Hope poly-PCA component 2 |  | 1.159 | 1.159 |
|  |  | (0.924 - 1.452) | (0.924 - 1.453) |
| Constant | 0.002*** | 0.002*** | 0.002*** |
|  | (0.000 - 0.046) | (0.000 - 0.046) | (0.000 - 0.047) |
|  |  |  |  |
| Observations | 496 | 496 | 496 |

Confidence intervals in parentheses

*** p<0.01, ** p<0.05, * p<0.1

Neither the first nor the second component of the hope index, constructed using polychoric PCA on the hope measures, was significant in the standard regressions; neither were they significant when included together in the same regression.
